# Rare functional missense variants in *CACNA1H*: What can we learn from Writer’s cramp?

**DOI:** 10.1186/s13041-021-00736-3

**Published:** 2021-01-21

**Authors:** Miaozhen Huang, Esther A. R. Nibbeling, Tjerk J. Lagrand, Ivana A. Souza, Justus L. Groen, Maria A. Gandini, Fang-Xiong Zhang, Johannes H. T. M. Koelman, Noam Adir, Richard J. Sinke, Gerald W. Zamponi, Marina A. J. Tijssen, Dineke S. Verbeek

**Affiliations:** 1 Department of Genetics, University Medical Center Groningen, University of Groningen, P.O. box 30 001, 9700 RB Groningen, The Netherlands; 2 Department of Clinical Genetics, Leiden University Medical Center, Leiden, The Netherlands; 3 Department of Physiology and Pharmacology, Hotchkiss Brain Institute, Alberta Children’s Hospital Research Institute, Cumming School of Medicine, University of Calgary, Calgary, AB Canada; 4 Department of Neurosurgery, Leiden University Medical Centre, Leiden, The Netherlands; 5 Department of Neurology and Clinical Neurophysiology, Academic Medical Center, University of Amsterdam, Amsterdam, The Netherlands; 6 Schulich Faculty of Chemistry, Technion–Israel Institute of Technology, Technion, Israel; 7 Department of Neurology, University Medical Center Groningen, University of Groningen, Groningen, The Netherlands

**Keywords:** Writer’s cramp, Focal dystonia, *CACNA1H*, Rare variants, Structural and functional analysis

## Abstract

Writer’s cramp (WC) is a task-specific focal dystonia that occurs selectively in the hand and arm during writing. Previous studies have shown a role for genetics in the pathology of task-specific focal dystonia. However, to date, no causal gene has been reported for task-specific focal dystonia, including WC. In this study, we investigated the genetic background of a large Dutch family with autosomal dominant‒inherited WC that was negative for mutations in known dystonia genes. Whole exome sequencing identified 4 rare variants of unknown significance that segregated in the family. One candidate gene was selected for follow-up, Calcium Voltage-Gated Channel Subunit Alpha1 H, *CACNA1H,* due to its links with the known dystonia gene Potassium Channel Tetramerization Domain Containing 17, *KCTD17,* and with paroxysmal movement disorders. Targeted resequencing of *CACNA1H* in 82 WC cases identified another rare, putative damaging variant in a familial WC case that did not segregate. Using structural modelling and functional studies in vitro, we show that both the segregating p.Arg481Cys variant and the non-segregating p.Glu1881Lys variant very likely cause structural changes to the Cav3.2 protein and lead to similar gains of function, as seen in an accelerated recovery from inactivation. Both mutant channels are thus available for re-activation earlier, which may lead to an increase in intracellular calcium and increased neuronal excitability. Overall, we conclude that rare functional variants in *CACNA1H* need to be interpreted very carefully, and additional studies are needed to prove that the p.Arg481Cys variant is the cause of WC in the large Dutch family.

Writer’s cramp (WC) is a task-specific focal dystonia that occurs selectively in the hand and arm during writing [1]. WC mainly affects the distal muscles of the arm but may spread to more proximal muscles and even to the non-dominant hand over time. The prevalence of WC—the most common form of a task-specific dystonia—is estimated at 2.7:100,000 [2]. Task-specific focal dystonia is thought to have a multifactorial aetiology, given its increased familial occurrence, but no clear family history is present in the majority of cases [3]. A few genes have been associated with either WC or focal dystonia [4], verifying a role for genetics in the pathology of task-specific focal dystonia.

In the present study, we aimed to identify the underlying cause in a Dutch family with genetically unexplained (no mutations found in known dystonia genes), dominantly inherited WC. The index patient (II-3; Fig. 1a) developed WC in his early twenties. At 50 years of age, he showed severe mobile flexion dystonia in the thumb of the right hand combined with extension in the wrist during writing, with an Arm Dystonia Disability Scale (ADDS) score of 3. His mother (I-2, Fig. 1a) noticed difficulties with writing from the age of 54. At examination at age 88, she showed a mobile, predominant flexion dystonia with tremor of the right hand (ADDS 3) during writing. The sister of the index patient (II-6, Fig. 1a) exhibited right-sided WC characterized by a tremulous writing pattern (ADDS 2) from the age of 36 years. Her son (III-7) suffered from WC from the age of 18 years. He showed dystonic posturing of the right thumb during writing. The daughter of patient II-3 is also reported to have difficulties with writing but has not been examined nor included in the genetic analysis.

**Fig. 1:**
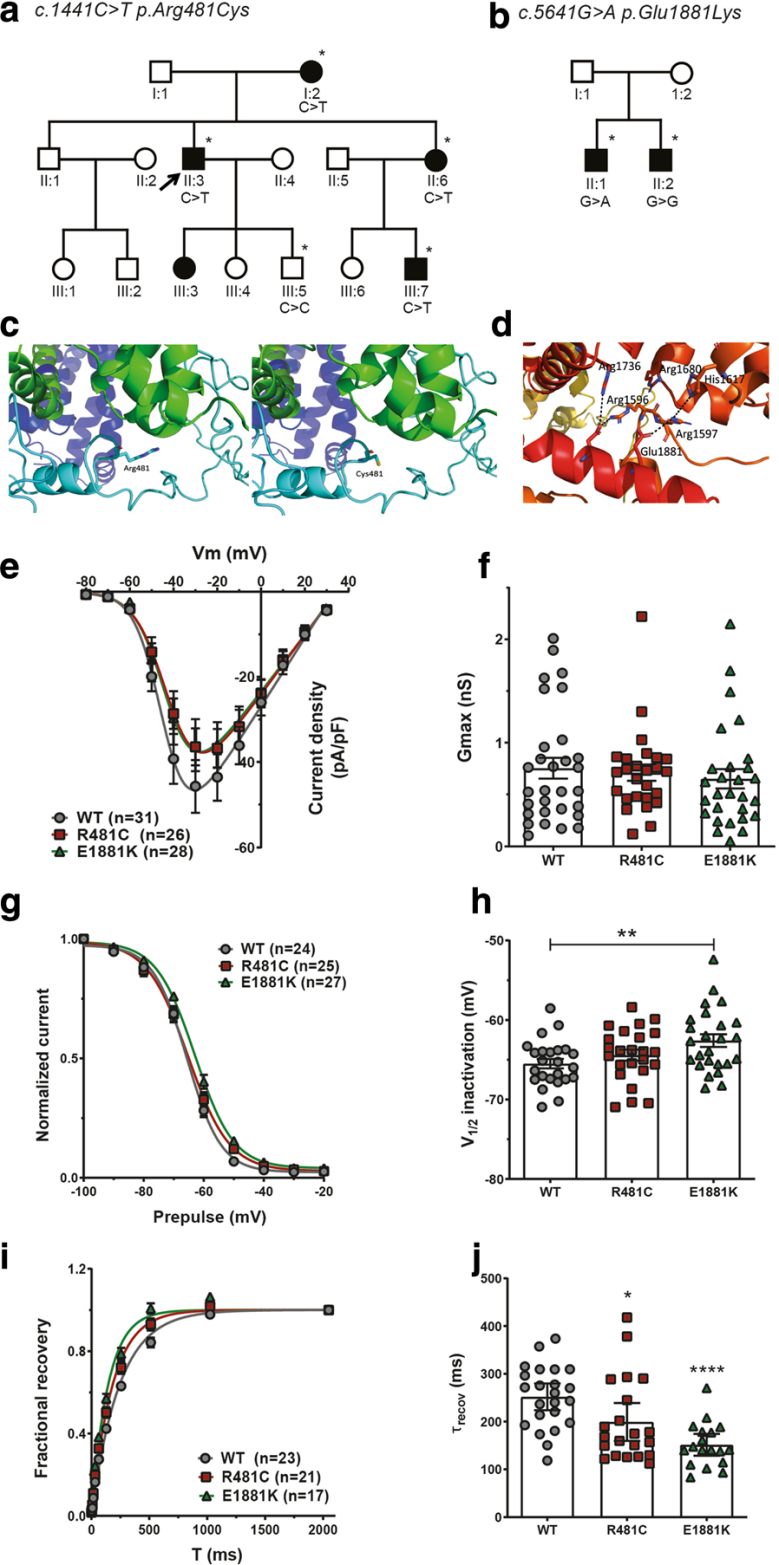
Segregation analysis and structural and functional characterization of two putative damaging missense variants in *CACNA1H.* **a**, **b** Pedigrees of the Dutch families with the c.1441C > T p.Arg481Cys variation in *CACNA1H* and the c.5641G > A p.Glu1881Lys variation, respectively. Open symbols indicate unaffected family members. Black symbols indicate affected members. Individuals marked with an asterisk were clinically examined and DNA was available for genetic testing. The index patient is marked by an arrow. **c** Arginine and cysteine at position 481 in the predicted structural model of Cav3.2. The introduction of a cysteine at position 418 might lead to destabilization of the bundle of α-helices (in green). **d** Glutamic acid at position 1881 is predicted to interact with adjacent arginines located at positions 1596 and 1597. **e** Average current densities (pA/pF) as a function of voltage in tsA-201 cells transfected with wild type (WT) Cav3.2, R481C and E1881K-mutant Cav3.2 channels. **f** Bar graph represents the corresponding maximum slope conductance G_max_. Values are represented as mean ± SEM. Solid lines are fits with the Boltzmann equation. **g** Mean normalized voltage dependence of steady-state inactivation of WT, R481C and E1881K channels. **h** Mean half-inactivation potentials determined via the Boltzmann equation from fits to individual steady-state inactivation curves. Asterisks denote statistical significance relative to wild type (**p = 0.0053, Student’s *t*-test). **i** Time course of recovery from inactivation. **j** Time constant of recovery from inactivation determined via individual fits of the recovery from inactivation curves. Asterisks denote statistical significance relative to WT Cav3.2 (*p = 0.0275, ****p < 0.0001, Student’s *t*-test)

After performing whole exome sequencing (WES) in II:3 and III:7, as described before [5], we discovered several rare missense variants shared between the two affected cases, but only 4 variants segregated with disease phenotype after Sanger sequencing (Table 1). All 4 variants exhibited Combined Annotation Dependent Depletion (CADD) Phred scores higher than 10 and were predicted to be probably damaging by Mutation Taster and/or Polyphen 2.0. Based on this data, these variants are classified as variants of unknown significance, and thus we could not define any of them as likely benign or likely pathogenic.

**Table 1:** Variants in genes co*-*segregating with the disease phenotype

Gene	Transcript	Transcript variant	Protein variant	gnomAD v3.1 (MAF)	CADD Phred score	Mutation Taster	Poly-Phen
*CACNA1H*	NM_021098	c.1441C > T	p.R481C	8/143316	18.2	PM	PrD
*GPER1*	NM_001039966	c.505C > T	p.R169C	2/143370	26.2	N.A	N.A
*SPTBN5*	NM_016642	c.8572C > T	p.H2858Y	Absent; present in dbSNP rs887835041	13.9	PM	PrD
*NUBP2*	NM_012225	c.296C > T	p.P99L	2/143346	22.9	DC	PoD

*MAF* minor allele frequency, *PM* polymorphism, *DC* disease-causing, *PrD* Probably damaging, *PoD* Possibly damaging, *N.A.* Not analysed. gnomAD browser accessed March 2020

Notably, an association between *CACNA1H*, which encodes a subunit of the neuronal voltage-gated T-type calcium channel Calcium Voltage-Gated Channel Subunit Alpha1 H, and dystonia has been proposed because a weighted dystonia gene co-expression network [6] directly connected *CACNA1H* to the known dystonia gene *KCTD17*, which encodes the protein Potassium Channel Tetramerization Domain Containing 17, leading to the assumption that both proteins function in the same signalling pathway. This was not the case for the other three candidate genes. Additionally, novel and rare variants in *CACNA1H* have been linked to childhood absence and idiopathic generalized epilepsy, familial hyperaldosteronism, amyotrophic lateral sclerosis and severe congenital amyotrophy [7–10]. Given that epilepsy overlaps with paroxysmal movement disorders such as focal dystonia [11], and the observation that *CACNA1H* functions in similar biological pathways as other known dystonia genes, we attempted to validate a role for *CACNA1H* in WC by screening the complete coding region of *CACNA1H* using a targeted array in a cohort of 82 genetically undiagnosed WC cases (both sporadic and familial). We identified 3 additional rare missense variants in *CACNA1H* in 3 WC cases: the c.5989G > A p.Ala1997Tyr variant predicted to be benign by various programs, the c.314T > G p.Val105Gly variant that was also detected in a patient with spinocerebellar ataxia type 3, and variant c.5641G > A p.Glu1881Lys, which was predicted to be damaging but did not segregate (Fig. 1b). This data reinforces that *CACNA1H* is relatively tolerant for rare missense variants, as confirmed by its gene constraint score of 1.17 (gnomADv3.1) [12].

To further investigate the consequence of rare missense variants in *CACNA1H*, we performed structural and functional analysis of the two putative damaging variants, p.Arg481Cys and p.Glu1881Lys. Structural analysis using the Protein Data Bank (PDB) entry 5GJW, showed that the p.Arg481Cys caused a likely loss of stability of an α-helix bundle and likely affects the α-helix bundle interactions in the interface with the main domain (Fig. 1c). Additionally, the presence of a cysteine at position 481 could lead to the formation of a disulphide bond with a native cysteine at position 847, which is located within the bundle, and this may cause conformational restraints that influence protein folding, stability and function. The introduction of the positively charged lysine at position 1881 due to the p.Glu1881Lys variant is likely to cause movement of the positively charged arginines at positions 1596 and 1597, changing the protein structure in this interface (Fig. 1d). Furthermore, we performed functional analysis of the mutant and wild type (WT) Cav3.2 channels in transiently transfected HEK tsA-201 cells, as done before [13]. Both variants did not change the conductance of the channel, as we observed a similar current density compared to WT Cav3.2 (Fig. 1e, f). However, the p.Glu1881Lys variant did cause a small, significant shift in the mean half-inactivation potential toward more positive potentials, and both variants led to an accelerated recovery from inactivation compared to WT Cav3.2 (Fig. 1g–j). This implies that Cav3.2 channels carrying the p.Arg481Cys and p.Glu1881Lys variants are less likely to inactivate and are available for re-activation earlier. This gain of function may lead to an increase in intracellular calcium and increased neuronal excitability [14, 15].

In summary, using WES, we identified 4 rare variants of unknown significance that segregated with the WC in the family. Given the established link between *CACNA1H* and the previously reported dystonia gene *KCTD17* and its link with paroxysmal movement disorders, we focused our additional studies on a putative role of *CACAN1H* in WC. Our follow-up work highlights that the need for caution in interpreting in silico predictions of rare missense variants in large genes like *CACNA1H* as damaging. We show that both the segregating p.Arg481Cys variant and the non-segregating p.Glu1881Lys variant very likely cause structural changes to the protein and lead to a similar gain of function of the Cav3.2 channel. Whether the p.Arg481Cys variant is the cause of disease in the large Dutch family remains to be proven, but our study corroborates that rare, functional missense variants in *CACNA1H* are quite common and may associate with numerous disorders, including WC.

## Data Availability

All data generated or analyzed during this study are included in this published article. The WES data is available upon request.

## Abbreviations

WC: Writer’s cramp
ADDS: Arm Dystonia Disability Scale
WES: Whole exome sequencing
CADD: Combined Annotation Dependent Depletion
PDB: Protein Data Bank
